# Towards natural mimetics of metformin and rapamycin

**DOI:** 10.18632/aging.101319

**Published:** 2017-11-15

**Authors:** Alexander Aliper, Leslie Jellen, Franco Cortese, Artem Artemov, Darla Karpinsky-Semper, Alexey Moskalev, Andrew G. Swick, Alex Zhavoronkov

**Affiliations:** ^1^ Insilico Medicine, Inc, Research Department, Baltimore, MD 21218, USA; ^2^ Biogerontology Research Foundation, Research Department, Oxford, United Kingdom; ^3^ Department of Biomedical and Molecular Science, Queen's University School of Medicine, Queen's University, Kingston, ON K7L 3N6, Canada; ^4^ Life Extension, Ft. Lauderdale, FL 33308, USA; ^5^ Laboratory of Molecular Radiobiology and Gerontology, Institute of Biology of Komi Science Center of Ural Branch of Russian Academy of Sciences, Syktyvkar, 167982, Russia

**Keywords:** geroprotector, metformin, rapamycin, deep learning, natural, nutraceutical, compound screening

## Abstract

Aging is now at the forefront of major challenges faced globally, creating an immediate need for safe, widescale interventions to reduce the burden of chronic disease and extend human healthspan. Metformin and rapamycin are two FDA-approved mTOR inhibitors proposed for this purpose, exhibiting significant anti-cancer and anti-aging properties beyond their current clinical applications. However, each faces issues with approval for off-label, prophylactic use due to adverse effects. Here, we initiate an effort to identify nutraceuticals—safer, naturally-occurring compounds—that mimic the anti-aging effects of metformin and rapamycin without adverse effects. We applied several bioinformatic approaches and deep learning methods to the Library of Integrated Network-based Cellular Signatures (LINCS) dataset to map the gene- and pathway-level signatures of metformin and rapamycin and screen for matches among over 800 natural compounds. We then predicted the safety of each compound with an ensemble of deep neural network classifiers. The analysis revealed many novel candidate metformin and rapamycin mimetics, including allantoin and ginsenoside (metformin), epigallocatechin gallate and isoliquiritigenin (rapamycin), and withaferin A (both). Four relatively unexplored compounds also scored well with rapamycin. This work revealed promising candidates for future experimental validation while demonstrating the applications of powerful screening methods for this and similar endeavors.

## INTRODUCTION

By 2030, the US Census Bureau projects that one in five people in the US alone will be over the age of 65 [1], a major risk factor for many of the most prevalent, costly, and devastating diseases of today, including cancer, cardiovascular disease, Alzheimer's disease, and Type II diabetes [2]. To offset the burden of this increase, efforts are underway to develop an anti-aging drug or other geroprotective intervention that could extend healthspan, lower disease rates, and maintain productivity in this age group.

Unfortunately, there are many roadblocks to such an intervention. While many aging mechanisms are now catalogued [3] and hundreds of databased drugs extend lifespan in animal models [4,5], approval and testing of new drugs in humans is slow, expensive, and prone to high failure rates. This is particularly true in longevity research and exacerbated by a lack of reliable aging biomarkers [6,7] other than disease itself [6,8]. Even if successful, to be used preventatively, anti-aging drugs face extraordinarily high safety and efficacy standards for approval [9].

One strategy to hasten the process has been the repurposing of existing, FDA-approved drugs that show off-label anti-cancer and anti-aging potential [10,11], and at the top of that list are metformin and rapamycin, two drugs that mimic caloric restriction [12].

Caloric restriction is a well-known intervention for extending lifespan across species [13], but has limited practical value in humans [14]. Mimetics of caloric restriction would theoretically exert its beneficial effects without actual reduction in caloric intake. Hallmarks of caloric restriction include reduced levels of circulating glucose and insulin as well as beneficial responses to these reductions in nutrient- and energy-sensing net-works, such as activation of AMP-activated protein kinase (AMPK) and inhibition of mammalian target of rapamycin (mTOR) [15]. The mTOR pathway is a particularly important growth pathway essential for early development but also potentially detrimental in later years if not suppressed, contributing to gero-conversion, cellular senescence, disease and decline [16]. Inhibition of the mTOR pathway slows conver-sion to senescence [16] and extends longevity across species, including *Saccharomyces cerevisiae* (yeast) [17], *Caenorhabditis elegans* (nematodes) [18,19], and *Mus musculus* (mice) [12,20–22].

Rapamycin and metformin, while distinct in clinical use, are both mTOR inhibitors and exhibit multiple anti-aging, anticancer, and anti-cardiovascular disease benefits [23].

Rapamycin (sirolimus) is an immunosuppressant used following renal transplantation, but also has life-extending properties in multiple animal models, including yeast [24], *Drosophila melanogaster* (fruit flies) [25], and mice [26,27], though effects can be sex and genotype-dependent [28]. In renal transplant patients, rapamycin has been shown to reduce cancer risk post-surgery [29–34]. It also has significant anti-cancer properties in mice [35–37]. While the extent to which its anticancer properties underlie its anti-aging effects and/or vice versa remains a point of discussion [15,38,39], as an anti-aging agent it has also been reported or theorized to protect against a number of other aging-related diseases in humans: cardio-vascular disease, osteoporosis, obesity, autoimmune disease and arthritis, macular degeneration, diabetes, Alzheimer's disease, and Parkinson's disease [16]. While rapamycin interacts with various nutrient signalling-related pathways, it acts primarily as an mTOR inhibitor, via direct inhibition of mTOR complex 1 (mTORC1) [23]. Analogs of rapamycin, or rapalogs (e.g. everolimus), are currently in use as anticancer drugs [40]. Also, mTORins, dual mTOR kinase inhibitors, are in development as anticancer agents, but much remains undetermined, such as proper dosage, toxicity, and adverse effects [15,38].

Like rapamycin, metformin is also an mTOR inhibitor, although indirectly so and via multiple mechanisms [41–45]. Metformin is a biguanide most renowned as the first-line treatment for type II diabetes and meta-bolic syndrome. It corrects hyperglycemia primarily by lowering hepatic gluconeogenesis but also by increasing insulin sensitivity and lowering levels of circulating lipids [9]. Its effects, however, appear to be pleiotropic, with benefits extending to a number of other age-related conditions in humans, including cancer [46,47] and cardiovascular disease [10]

In animal models as well, multiple beneficial effects of metformin have been reported across species with varying anticancer and prolongevity effects, including AMPK-mediated improvements in cutaneous wound healing [48]. Results, however, depend on dosage, sex, and age at onset of treatment [49–53], factors relevant to widescale, prophylactic metformin use in humans [49,50].

Metformin's mechanisms of action have been extensively studied but are complex and remain only partially understood. Although metformin inhibits mTOR [43-45], its primary mode of action may be inhibition of mitochondrial complex I [54–62]. This action leads, among other things, to beneficial changes in cellular energy status and activation of AMPK [51,59, 62–66], a cellular energy sensor with a broad range of downstream effects on cellular function [67]. Through a combination of AMPK-dependent and -independent mechanisms [68], metformin influences a number of signaling pathways, including IGF-1 [69], hepatic sirtuin 1 (SIRT1) [70–73] and mTOR complex 1 (mTORC1) [74], that contribute directly or indirectly to its clinical response and multiple anticancer effects.

Taken together, rapamycin and metformin are promising candidates for life and healthspan extension; however, concerns of adverse side effects have hampered their widescale adoption for this purpose. While short term rapamycin use is considered safe, it has been reported to be associated with more adverse events than cyclosporin A in renal transplant patients, including wound complications, mouth ulcers, diarrhea, hypokalemia, bronchopneumonia, and proteinuria and higher discontinuation rates (28.2% vs 14.9%) [75–77]. In addition, chronic rapamycin use can lead to hepatic gluconeogenesis, insulin resistance, and severe glucose intolerance in rats [78], impaired glucose tolerance in mice [79], and even diabetes in male mice [80]. While rapamycin-induced diabetes is argued to differ from true type II diabetes [81], rapamycin may require pairing with metformin to counter induced hyper-glycemia [40]. Metformin, while relatively safe, is poorly tolerated in one fourth to one half of patients due to gastrointestinal side effects [82], although prelimi-nary findings suggest these can be alleviated in some with an extended-release form of the drug [83]. Metformin also carries a slight risk of lactic acidosis in certain individuals [84–86]. Interestingly, rapamycin lowers lactate production, so may buffer this risk [87]. Metformin and rapamycin in combination may have additional benefits; *in vitro* they potentiate chemo-therapy with mitotic inhibitors while protecting normal cells [41]. One suggestion has been varying dosage schedules and combinations of rapamycin with metformin and five other anti-aging compounds per individual to reduce side effects [40]. However, the best preventative, widescale intervention would be one for which risk is negligible.

Given the urgency of the present need for anti-aging, disease preventive interventions, it may be beneficial to look to natural alternatives, such as nutraceuticals, that would be safe enough to administer widely with little to no risk of harm and with fewer regulatory hurdles than drugs. Nutraceuticals have received considerable attention in recent years for potential roles in preventing or treating a number of age-related diseases [88].

In this work, we initiate an effort to identify safe, natural alternatives to metformin and rapamycin. Our work is done entirely *in silico* and entails the use of metformin and rapamycin transcriptional and signaling pathway activation signatures to screen for matches amongst natural compounds. We have shown previous-ly that the transcriptional signature of a given drug response, disease state, or other physiological condition, when mapped to the signalome, can be useful for biomarker development [89–91] and drug screening [7,92,93]. Transcriptional signatures have been suggested by others as well for aiding in biomarker development [94], cancer drug screening [75] and repositioning [11], and diabetes management [95].

The transcriptional signature of metformin is particularly well-suited to this type of analysis, as it includes thousands of AMPK-dependent and AMPK-independent changes in gene expression related to a diverse set of signaling pathways [96]. AMPK itself acts in part by directly and indirectly regulating metabolic gene expression when activated [97]. Metformin's transcriptional signature also shows considerable similarity to the gene expression signature of long term caloric restriction [98,99,49], which is thought to play a role in mediating its effects on lifespan [100,101].

Gene expression data is in general a highly valuable resource that is still underutilized in drug discovery. With the public banking of data such as the LINCS project resulting in large repositories of cellular signatures of drug responses and disease states, large-scale screening, signalome analysis, and deep learning can be employed at little cost to make new discoveries [102]. Yet due to the size, difficulty in cross-platform analysis, and high dimensionality of microarray datasets, much information remains unparsed.

To overcome and even exploit these challenges, we have developed bioinformatic methods including Oncofinder [92,103], Geroscope [93], and in silico Pathway Activation Network Decomposition Analysis (iPANDA) [104], which extract robust, biologically relevant pathway activation signatures from the data by combining various elements of previous approaches. The iPANDA method in particular was recently shown to outperform other methods in cross-platform micro-array analysis, noise and dimensionality reduction, and production of robust sets of biomarkers and reliable pathway signatures [93]. Illustratively, it was used successfully to identify biomarkers for breast cancer subtypes by stratifying samples by pathway activation [104], however it has many other potential applications, including drug discovery and drug mimicry, as we will demonstrate herein. We are currently using iPANDA in several other applications, including mapping the transcriptional signature of senescence and screening for novel senolytics, drugs that would selectively eliminate senescent cells [8]. We have also previously developed deep learning methods involving training of deep neural networks (DNNs) to recognize trans-criptional signatures and pathway activation signatures of drugs or disease states from microarray data or to predict adverse effects [93].

In the present study, we apply these methods to screen for nutraceuticals that mimic metformin and/or rapamycin. Using LINCS perturbation data, we reduce a list of over 800 natural compounds to a shortlist of candidate nutraceuticals that show both similarity to the target drugs and low adverse effects profiles [93]. We then discuss the top candidates in light of shared mechanisms and previously reported anticancer and other health benefits that may deem them particularly promising for future experimental validation.

## RESULTS

**Figure 1:**
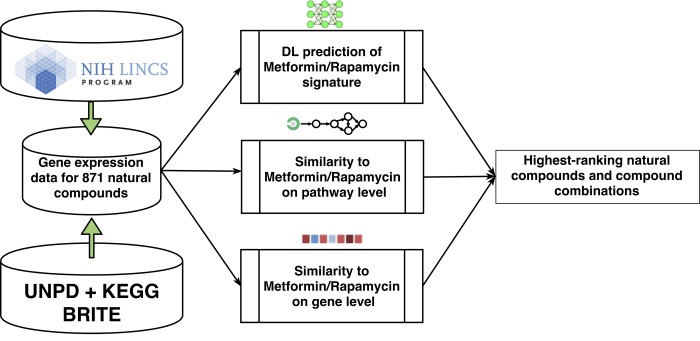
Workflow diagram showing multi‐level analysis for screening and ranking nutraceuticals that mimic metformin and rapamycin in transcriptional and pathway activation response. A subset of 871 LINCS compounds were selected from the UNPD and KEGG BRITE databases. Perturbations with those compounds in cancer cell lines were compared with perturbations with metformin and rapamycin to estimate similarity at the gene and pathway level and deep learning techniques were employed to recognize the transcriptional signature of metformin and rapamycin and screen for matches amongst the LINCS compounds.

To screen for potential candidate nutraceuticals, we used gene expression data from the Library of Integrated Network-based Cellular Signatures (LINCS) L1000 dataset to investigate similarity to metformin and/or rapamycin at the gene and pathway level (Figure 1). We employed several complementary approaches, including conventional statistical methods, pathway scoring-based methods, and training of deep neural networks (DNN) for signature recognition. Additionally, to evaluate potential adverse effects of top-scoring natural compounds we utilised a set of deep learned predictors, trained on drug-induced trans-criptional response data. One important attribute of natural compounds we also looked closely at was GRAS (Generally Recognized As Safe) status and safety data.

### Selection of natural compounds for screening

Prior to analysis, we filtered the LINCS dataset for compounds of natural origin by combining the compound lists from UNPD [105] and KEGG BRITE [106] databases and using the resulting list to select compounds in the LINCS dataset. In total, this resulted in 871 natural compounds with transcriptional response data across various times, concentrations, and cell lines. We utilized all available gene expression profiles for each compound, including metformin and rapamycin.

### Deep learning-based scoring of compounds at gene level

**Figure 2:**
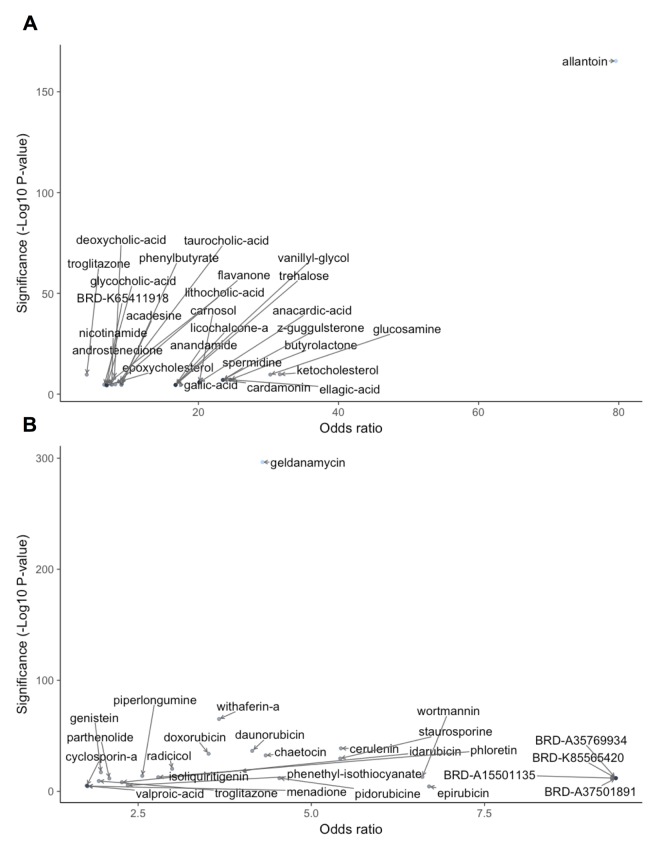
DL‐based similarity to metformin (**A**) and rapamycin (**B**). Significance of natural compound was determined as the ‐log10(p‐value) and odds ratio for compound according to Fisher's exact test performed on the DNN output for each perturbed sample. Only compounds with ‐log10(p‐value)>4 and odds ratio > 1 are shown.

For similarity scoring, we first used deep learning to train binary classifiers to recognize perturbations similar to metformin or rapamycin in transcriptional signature. A five fold cross-validation classifier for metformin and rapamycin achieved an F1-score of 0.725 and 0.905 and Matthews correlation of 0.705 and 0.896, respectively. Each sample corresponding to perturbation with a natural compound was run through each DNN classifier and assigned a probability. We used a threshold of 0.5 to determine the significant hits and then performed a Fisher's exact test to estimate the statistical significance for each compound (Figure 2, Supplementary Tables 1 and 2).

The compound exhibiting the highest similarity to metformin according to the metformin classifier (Fig. 2A) was allantoin, a key beneficial compound in yam (*Dioscorea spp*.). Like metformin, allantoin is a guanidinium derivative with anti-hyperglycemic effects [107,108]. It is an important metabolic intermediate of purine metabolism in many species across Eukarya and Bacteria domains [109,110]. Being a guanidinium derivative, allantoin is similar to metformin in structure and has been shown to induce glucose lowering effects via imidazoline I-2 receptors [107,108]. Other top hits for metformin included glucosamine, a compound used in the treatment of osteoarthritis [111,112], and car-damonin, a member of the anti-inflammatory chalcones found in plant-based foods [113], which inhibits mTOR and exhibits antitumor effects *in vitro* and *in vivo* [114].

With the rapamycin classifier, the most significant hit was geldanamycin (Fig. 2B). Geldanamycin is an anti-biotic belonging to Ansamycins family and targets the ADP/ATP binding site of heat shock protein 90 (Hsp90). Similarly to rapamycin, it has been shown to suppress the mTOR pathway through inhibition of the interaction between Hsp90 and RAPTOR [115]. Interestingly, the second most significant hit was withaferin A, which aligned with our subsequent results of gene- and pathway-level scoring for metformin and rapamycin, respectively. Other compounds with significant similarity to rapamycin according to the DNN classifier included another Hsp90 inhibitor, radicicol, several members of the anthracyclines antibiotic family used in cancer treatment (dauno-rubicin, idarubicin, doxorubicin, epirubicin) [116], cerulenin, a fatty acid synthase inhibitor with potential anticancer effects [117], chaetocin, being investigated as a histone lysine methyltransferase inhibitor [118,119], phloretin, an anti-tumor agent found in plant-based foods that shows effectiveness in inducing apoptosis in human lung cancer cells [120] and staurosporine, which also exhibited metformin simila-rity in subsequent results. The highest odds ratios were observed with four relatively unexplored compounds (BRD-A35769934, BRD-K85565420, BRD-A15501135, BRD-A37501891). Notably, 24 of 24 profiled samples for each of these compounds reached statistical sig-nificance.

### Similarity at gene and pathway level

We next determined gene-level similarity of each compound to metformin and rapamycin using conventional statistical methods. First, this involved comparing each distinct time- and concentration-specific compound perturbation measured across various cell lines to corresponding DMSO-treated reference samples. We performed differential gene expression analysis to determine statistically significantly perturbed genes. Then, to screen for compounds with high similarity to metformin or rapamycin in terms of individual gene expression changes, we used Fisher's Exact Test to directly compare all metformin or rapamycin perturbations to individual perturbations of other natural compounds (Supplementary Table 3).

To determine pathway-level similarity, we applied the iPANDA algorithm [104] to acquire pathway activation profiles for the same set of individual perturbations. For each compound, perturbation pathway activation scores (PAS) were calculated for 378 pathways. Similarity of pathway activation signatures of natural compounds to metformin and rapamycin was evaluated by the number of commonly up- and down-regulated pathways (Supplementary Table 4).

**Figure 3:**
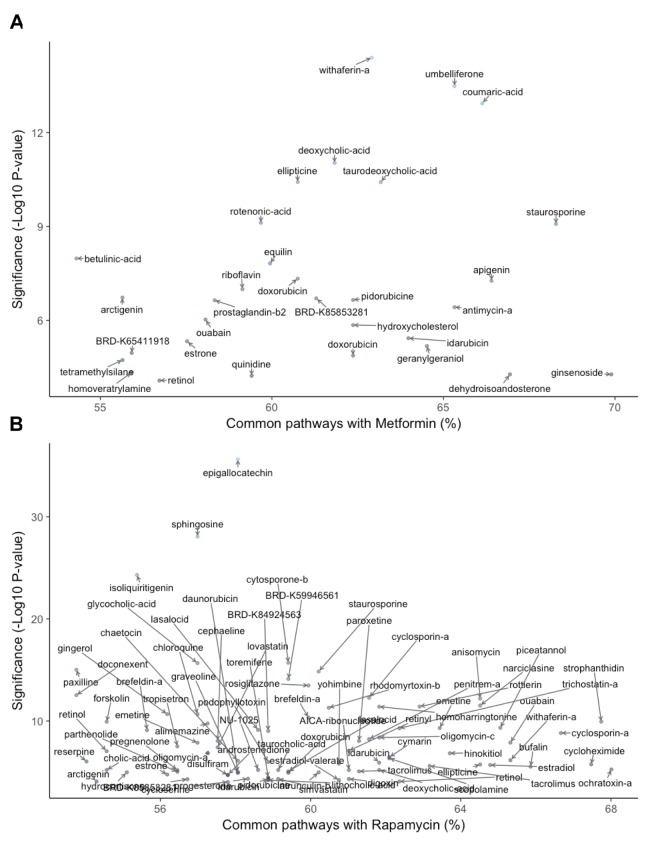
Gene‐ and pathway‐level similarity to metformin (**A**) and rapamycin (**B**). Significance of natural compound was determined as the ‐log10(p‐value) of the most significant perturbation of compound according to Fisher's exact test. Percentage of common pathways designates the amount of pathways that have the same direction of the change as Metformin. Only compounds with ‐log10(p‐value)>4 and over 50% of common pathways are shown.

Combined results of gene- and pathway-level analysis are depicted in Figure 3. Gene-level analysis of similarity to metformin (Fig. 3A) showed that the most significant perturbation was associated with withaferin-A, a steroidal lactone that exhibits antidiabetic and anticancer properties [121] Pathway-level scoring, on the other hand, demonstrated ginsenoside Rc, a compound isolated from ginseng, to be the top hit. Other compounds at the top of the list for significant gene- and pathway-level similarity to metformin included umbelliferone, a coumarin with antihyper-glycemic, anti-inflammatory, and antitumor properties [122], coumaric acid, the p- isomer of which shows immunosuppressive, anti-inflammatory, and anti-diabetic properties [123,124], staurosporine, a kinase inhibitor with promising antitumor properties but poor selectivity [123–125], bile acids, which have been shown to have anti-cancer properties and specifically anti-hypoxic tumor effects [126], and ellipticine, a plant-derived compound with significant anticancer effects but issues with toxicity [127].

For rapamycin (Fig. 3B), the most significant hits at the gene level were epigallocatechin gallate (EGCG), a compound underlying the aging-related benefits of green tea, including protection against cancer, cardiovascular events, and UV-mediated skin aging [128], sphingosine, the precursor to sphingosine 1-phosphate, a second messenger implicated in several diseases, including multiple sclerosis, sepsis, cancer, and cardiovascular disease [129], and isoliquiritigenin, a compound shown to act as an anticancer, anti-cardio-vascular disease, antioxidant, antimicrobial, hepato-protective, and immunoprotective agent [130]. A num-ber of other compounds were highly similar to rapamycin at the pathway level. These included strophanthidin, a compound recently identified in a similar LINCS screening as being likely to reverse cancer-related gene expression, which was validated in the liver hepatocellular carcinoma (LIHC) cell line [75], cyclosporin A, an immunosuppressant alternative to rapamycin following transplantation [75,76] cyclo-heximide, a highly toxic protein synthesis inhibitor used primarily in basic research, including cancer research [131], ochratoxin A, a potentially carcinogenic myco-toxin found and regulated in a wide variety of foods [111], and, notably due to its gene-level similarity to metformin above, withaferin A.

### Effective combinations of natural compounds

Often, natural remedies with proven effectiveness consist of one or several plant species which can account for hundreds of natural compounds [132]. Accumulating evidence suggests that a combination of several compounds targeting multiple pathologic signaling circuits might be more advantageous than single agent treatments [133–137]. Examples of syner-gistic anti-aging effects of drug combinations with different targets have been reported [37,138]. This is particularly relevant to natural compounds with GRAS status, since the likelihood of serious adverse reactions is low.

For these reasons, we also estimated the similarity of different combinations of natural compounds to metformin. This required us to predict the trans-criptional response after perturbation with a given combination of compounds. We chose to do this on the pathway level and to calculate the combinatorial response as the sum of individual PAS values cor-responding to individual perturbations. We fully considered that additive effects on a pathway may be limited and other types of effects (e.g. synergistic, com-petitive, etc.) may be at play. Our rationale for assuming additivity was required for simplification, but we tested the additivity assumption for its predictive value with an external dataset and the results supported the approach. We used external dataset E-MEXP-3192 (http://www.ebi.ac.uk/arrayexpress Supplementary Figure 1) [139], where the pathway activation signature of two compounds, retinoic acid and lapatinib, was explored, both individually and in combination, to predict their combinatorial drug effects by taking the sum of individual PAS values. Results, at least in the case of these two compounds, showed high similarity between the predicted and actual combinatorial pathway activation signatures, supporting the use of PAS additivity in this context (Supplementary Figure 1).

To investigate whether any of the natural compound combinations would produce better similarity scores than each compound independently, we selected four compounds with known beneficial effects and good safety profiles: withaferin-A, ginsenoside, apigenin and gamma linolenic acid (GLA).

We used our previously established database of aging-associated pathways and calculated PAS values for each compound (Supplementary Table 5). Then we devised all possible combinations of these compounds and estimated the resulting pathway activation as the sum of PAS values of individual compounds. Each of the combinations was compared to the profile of metformin and Pearson correlation coefficient was used as a similarity metric (Supplementary Table 6). Combina-tions outperformed the individual compounds, with similarity of the top 10 combinations ranging from 0.73-0.80 (Supplementary Table 6). As an example, we selected a combination of three nutraceuticals with high similarity to metformin, good safety profiles, and/or previously reported anti-aging, anticancer, or anti-disease potential: ginsenoside, GLA, and withaferin A. Overall pathway level similarity between metformin and the top combination of nutraceuticals is depicted in Sup-plementary Figure 2. Pathways with shared activation between metformin and the combination of these three compounds and each compound individually are shown in Supplementary Figure 2; the most significant of these were upregulation of JNK, cAMP, AKT, MAPK, ERK, and ILK pathways and down-regulation of ubiquitin proteosome signaling. To investigate whether similarity varied among met-activated, met-neutral, and met-inhibited pathways, we also examined correlations between metformin and the nutraceuticals and nutra-ceutical combination among these groups, with a designated threshold of 1 or −1 to define met-activated or met-inhibited pathways, respectively; results showed the strongest correlations with pathways inhibited by metformin (Supplementary Table 7).

### Deep learning-predicted adverse effects

Additionally, to estimate the safety of investigated natural compounds we utilized our deep learned adverse effects prediction approach [93]. For every sample corresponding to perturbation with a natural compound, we ran an ensemble of 305 predictors each correspond-ing to a particular side effect category. Resulting probabilities were averaged for each side effect of each natural compound. Then, to estimate the overall adverse effects prediction of a compound, we calculated mean probability across all adverse effects and the number of adverse effects with probability >0.5 (Supplementary Table 8).

Interestingly, rapamycin was near the top of the list of compounds with the highest probability of adverse effects, with a maximum mean probability of 0.41 across all potential adverse effects and 134 total effects categories for which probability exceeded 0.5. Of these, the top ten adverse effects categories included cardiac and vascular, lipid, testicular and epididymal, skin, general, immunodeficiency, obstetric and gynecological, eye, neurological, and vascular/hyper-tensive, all with probabilities >0.9. Metabolic (0.86) and glucose/ diabetic (0.75) effects probabilities were also high for rapamycin. Other compounds with high mean adverse effects probabilities included anthra-cycline antibiotics, oligomycin-c, tacrolimus, paroxetine, benzethonium, wortmannin and triptolide. The safest compounds, on the other hand, with <3 significant adverse effects categories and mean overall probabilities <0.05, turned out to be the compounds with highest odds ratios for rapamycin similarity scoring (BRD-A35769934, BRD-K85565420, BRD-A15501135, BRD-A37501891) as well as tert-butylhydroquinone, lanatoside-c, syringic acid, morin, niacin and gossypetin (mean probabilities <0.10, 11 or fewer significant adverse effects categories). Metformin was predicted to have relatively few adverse effects, as well, with mean probability 0.2 and 25 significant adverse effects categories.

We then searched the adverse effects table against the list of candidate compounds selected above for metformin and rapamycin similarity to investigate predicted adverse effects. For metformin-like compounds, we found the following mean adverse effects probabilities and number of adverse effects categories: withaferin A (0.14, 52), staurosporine (0.17, 126), ginsenoside (0.25, 29), umbelliferone (0.24, 19), ellipticine (0.17, 69), allantoin (0.14, 28), glucosamine (0.25, 58), cardamonin (0.26, 66). For rapamycin-like compounds, we found similar probabilities and numbers of categories: ECGC (0.25, 31), sphingosine (0.20, 46), isoliquiritigenin (0.23, 88), strophanthidin (0.17, 38), cyclosporin A (0.26, 62), ochratoxin A (0.19, 39), geldanamycin (0.20, 57), radicicol (0.20, 87), cerulenin (0.22, 49), and chaetocin (0.09, 23).

## DISCUSSION

In this work, we introduce a rapid, low-cost route to drug mimicry via screening public gene expression datasets for compounds with shared transcriptional and signaling pathway activation signatures. The methods we employ [104] combine and outperform previous methods for pathway activation scoring and capitalize on vast, valuable but underutilized public repositories of microarray data, overcoming significant analytical challenges that have previously hindered their wide-scale use.

In an application of these methods, we focused on mimicry of metformin and rapamycin, seeking nutraceuticals that could preserve their anti-aging and disease-preventive potential while being better suited for wide-scale prophylactic use.

One of the most significant findings was withaferin A, one of only two only compounds topping the list for similarity to both metformin and rapamycin. Withaferin A was the top-scoring compound for gene-level similarity to metformin using the conventional statistical approach and also displayed significant pathway- and gene-level similarity to rapamycin using both the pathway activation approach and the deep learning approach. Withaferin A is a steroidal lactone derived from members of the Solanaceae family (e.g. Acnistus arborescens and Withania somnifera), commonly used in Ayurveda (traditional Indian medicine) for arthritis and menstrual disorders. Mounting evidence in rodent and cell-culture models indicate that it is an anti-diabetic, anti-obesity and anti-cancer agent with potent anti-oxidative, anti-inflammatory, anti-proliferative, apoptosis-inducing and leptin-sensitizing properties [121].

Mice with diet-induced obesity (DIO) have seen 20-25% reductions in body weight as a result of withaferin A treatment [140], as well as a decrease in obesity-associated pathology, e.g. hepatic steatosis. Withaferin A also has beneficial effects on glucose metabolism that are independent of its leptin-sensitizing effect.

Many of its anticancer properties result from its ability to inhibit cell proliferation and decrease glucose utilization, glycolysis and tricarboxylic acid (TCA) cycle intermediates [141]. Additionally, it has been found to be a potent inhibitor of angiogenesis. It inhibits cell proliferation via inhibition of cyclin D1 expression, as well as inhibition of NF-kappa B, which is thought to occur via interference with the ubiquitin-mediated proteasome pathway [142], as suggested by increased levels of polyubiquitinated proteins in cancer cells following treatment with withaferin A. It has also been found to selectively induce cell death in multiple types of tumor cells [143,144]. Its anticancer effects are mediated through modulation of a number of pathways, including inhibition of Notch 1 [145], inhibition of STAT3 activation [146–148], downregulation of the MTOR signalling components pS6K and p4E-BP1 [145], downregulation of the prosurvival pathway Akt/NF-kappaB/Bcl-2 [145], induction of c-Jun-NH(2)-kinase-mediated apoptosis [145], induction of apoptosis via upregulation of Bim, t-Bid, caspase-8, and DR5 [149], suppression of constitutive and IL-6-induced phosphorylation of STAT3 (on Tyr705) and consequent down-regulation of the STAT3 regulated genes Bcl-xL, Bcl-2, cyclin D1 and survivin [150], inhibition of heat shock protein 90 [151], downregulation of COX-2 and iNOS by blocking NF-κB activity [121], and down-regulation of TNF-a [152].

Withaferin A was one of three compounds we included in the combination explored for metformin similarity. The other two were ginsenoside and GLA, which also demonstrate anti-aging, anticancer, and anti-disease potential in a number of studies.

Ginsenoside was the most similar compound to metformin at the pathway level. Used in traditional Chinese medicine, ginsenosides comprise a group of over 150 naturally occurring compounds isolated from plants of the Panax species (ginseng) [153]. Although the family is relatively diverse in term of chemical structure, most of its members share similar properties. A wide variety of benefits have been reported [153], including anticarcinogenic [154–157], immuno-modulatory [157–161], anti‐inflammatory [162], anti-allergic [163–165], antiatherosclerotic [166], anti-hypertensive [167,168], antidiabetic [169], anxiolytic [170,171] and antidepressant effects [172]. Ginseno-sides activate AMPK [154,156,169,173] PI3K [169] and SirtI [169,174] pathways, promoting autophagy [154–156] and apoptosis [154–156] in cancer cells.

Another clear standout for metformin similarity was revealed by the DNN classifier, and that was allantoin, one of the active compounds mediating beneficial effects of yam. Yam powder, yam extract, and allantoin have been shown to improve B-cell function in maintaining insulin and glucose in a rat model of Type II diabetes, with antioxidative effects as well, improved lipid profiles, and increased release of glucagon-like peptide 1 (GLP1) [175]. In another study using the same rat model of diabetes, allantoin lowered plasma glucose levels by increasing ß-endorphin secretion, increasing GLUT4 expression, and increasing glucose uptake [108,176,177].

Overall, the most remarkable aspect of the metformin results was that, like metformin, several of the compounds scoring high in similarity exhibit glucose-lowering properties (withaferin A [121], umbelliferone [122], and allantoin [107,108]) or anti-inflammatory effects (glucosamine [111,112] and cardamonin [113]) in previous studies, and almost all of the top hits have shown anticancer potential, including withaferin A [141–144], ginsenoside [154–157], umbelliferone [122], staurosporine [123–125], bile acids [126], ellipticine [127], cardamonin [113], and allantoin [177]. This not only lends preliminary support to the validity of our methods, but also adds support to the evidence of metformin-like health benefits with these compounds.

Scoring for rapamycin overall revealed a larger number of significant hits compared to metformin, but more variation in the range of known effects, from beneficial to highly toxic. These also included several unnamed, novel candidates. Four of these relatively unexplored compounds (BRD-A35769934, BRD-K85565420, BRD-A15501135, BRD-A37501891) were the most significant in similarity to rapamycin and were also top-ranking in terms of safety, with extremely low probability of predicted adverse effects. These would be excellent novel candidates for characterization and validation in future work.

Like the metformin DNN classifier, the rapamycin classifier also revealed a clear standout amongst the compounds for rapamycin similarity, geldanamycin. Geldanamycin is an inhibitor of Hsp90 [178], which is an oncogenic target molecule overexpressed in many tumors [115,179]. Geldanamcyin is an inhibitor of mTOR signaling as well [115] While initially promising as an potent anticancer agent [115,179,180], its hepatotoxicity has precluded its clinical use [180,181]; however, several less toxic derivatives have been developed [182], with 17AEP-GA and 17DMAG recently demonstrating growth suppression of multiple myeloma cells similar to geldanamycin [181]. Geldanamycin analog development is still an active area of research [182–185], with other analogs being recently shown to be effective against breast cancer cells [182,185]. In addition to geldanamycin, at least two of the other rapamycin hits in this study, radicicol and EGCG are also Hsp90 inhibitors [183,184]. Recently, a radicicol derivative, NW457, was shown to be effective against colorectal cancer both *in vitro* and *in vivo* [186].

Many of the other top hits for rapamycin show anticancer effects, including anthracyclines [116], cerulenin [117], isoliquiritigenin [130], strophanthidin [75], ECGC [128], phloretin [120], staurosporine [123–125], and withaferin A [141–144]. Several of the rapamycin-like compounds identified are known to modulate mTOR signaling. These include geldana-mycin, which suppresses mTOR phosphorylation of downstream protein regulators [115], phloretin, a common flavonoid capable of inducing cell cycle arrest and apoptosis in part via suppression of AKT/PI3K/mTOR cascades [187], and isoliquiritigenin, another flavonoid that induces autophagic and apoptotic cell death in cancer cells via mTOR signaling [188]. Thus, like metformin, many of the compounds identified as being similar to rapamycin in transcriptional signature have been previously shown to have rapamycin-like properties. Other rapamycin-like compounds identified have mTOR-independent mechanisms, such as chae-tocin, a histone H3K9 methyltransferase inhibitor [119].

Finally, rapamycin had a remarkably high number of predicted adverse effects with our methods and significant similarity to at least two compounds known to be toxic, ochratoxin A and cycloheximide, although these toxic compounds were not predicted to have a wide variety of adverse effects (cycloheximide did score particularly high (0.86) in the toxicity category, however, as did strophanthidin (0.93)). This under-scores the need to look for rapamycin alternatives, and also raises interesting questions about the common (and distinct) mechanisms between rapamycin and the wide variety of rapamycin-like compounds, both beneficial and toxic.

The adverse effects prediction also enabled us to have a closer look at overall predicted safety of compounds of interest and likelihoods of specific adverse effects. None of the compounds discussed as similar to metformin or rapamycin stood out as extremely likely or unlikely to cause a wide variety of adverse effects; most scored in the low-moderate range, although this does not fully reflect the severity or importance of any one given adverse effects category for a given compound. Literature-based assessments of safety were also helpful; while several compounds are known to be toxic as noted, others are known to be relatively safe compounds found in plant-based foods, such as cardamonin and ECGC, or used in traditional medicine, such as withaferin A and ginsenosides. Safety in a preventative, chronic use context for each compound would have to be independently evaluated. Also, while there were no standout metformin-like candidates with an absence of gastrointestinal adverse effects, there were several rapamycin-like candidates with low likelihood of glucose/metabolic adverse effects, including withaferin A and ECGC. Perhaps the most notable compounds were the four unnamed compounds with similarity to rapamycin; their novelty, extremely low number of predicted adverse effects, including glucose/metabolic effects, and extremely high odds ratios for rapamycin similarity make them particularly intriguing candidates.

The *in silico* approach, while time- and cost-saving, does require several considerations in light of its role as a first-pass screening tool. First, and most importantly, the health-extending and adverse effects of all candidate nutraceuticals or other compounds will still require investigation and validation *in vitro* and in other cell lines, followed by validation *in vivo* in humans. This is particularly important in the case of nutraceuticals, as wide variation in their bioavailability and metabolism is a significant factor influencing the degree to which the predicted effects actually manifest *in vivo* [189]. Secondly, our approach hinges entirely upon the biological relevance of the short term (<48 hours) transcription-level response to a drug, and as such does not account for post-transcriptional and post-translational effects on a given pathway or long term changes, which may be biologically or clinically more important [190]. That said, numerous studies have demonstrated the value in using such expression signatures in the characterization of drug response [191].

Thus, while it cannot be overstated that our results will require validation, this work reduces a list of over 800 natural compounds to a manageable shortlist of a few strong candidates for metformin and rapamycin mimicry, substantiated by their similarity to the target drugs in transcriptional response. Several of these compounds are unnamed, novel candidates. Many of the others have known anticancer or other beneficial effects and now are demonstrated to share common cellular signatures with two known anticancer, anti-aging drugs, thus supporting previous findings and further investigation into their potential benefits. That so many compounds with anticancer and other health benefits share common transcriptional signatures raises interesting questions about what pathways are shared and distinct and which shared pathways are most critical to their beneficial effects. This has not only direct practical value in a narrow sense with the search for metformin and rapamycin mimetics, but has broader usefulness for any number of applications in drug discovery. If widely adopted, our approaches have the potential to significantly expedite drug development timelines, reducing cost by offering a viable and biologically-relevant means of screening and ranking compounds prior to *in vitro* studies and, since screening is based on human data, possibly in place of animal models. Improving our ability to predict the actions of a nutraceutical or drug in humans will give *in silico-*based approaches enormous utility in streamlining drug discovery, repurposing and development in the years to come.

## METHODS

### Transcriptomic data

To obtain transcriptomic and signaling pathway activation signatures, we utilized transcriptional response data provided by LINCS Project (http://www.lincsproject.org/). To obtain a list of natural compounds present in the LINCS data set we used the UNPD database of natural compounds [105] in combination with 3 compound classification categories derived from KEGG BRITE Database [106]: “Phytochemical compounds”, “Phytochemicals used as drugs” and “Natural toxins”. The natural compound list was then compared to the list from the LINCS data set and 871 compounds were identified.

For each of these compounds, we extracted the level 3 (Q2NORM) gene expression data for each available cell line perturbed with each concentration of compound independently for all available timepoints. In the pathway-level analysis, for each case sample group perturbed with a compound, we generated a reference group consisting of samples perturbed with DMSO that came from the same RNA plate as samples from the case group. We analysed transcriptional response to perturbation with metformin, rapamycin, and a number of nutraceuticals as assayed in various cancer cell lines.

### Differential expression

For transcriptome data, a limma test of differential gene expression was used. Each set of differentially expressed genes was ordered according to the following measure, which takes into account both the magnitude and statistical significance of the effect: FC * max(0, -log(p-value)), where FC is fold-change of gene expression between groups and p-value represents the result of limma test.

### Gene level similarity to metformin/rapamycin

A statistically motivated score estimating the similarity of a compound was designed. Significantly up- or down-regulated genes were defined as those with an FDR-adjusted p-value of <0.01. A Fisher's exact test was used to measure the association between two characteristics of each gene: being significantly down-regulated following metformin/rapamycin treatment and being significantly downregulated following treatment with each investigated compound in our compound library. The same test was performed for upregulated genes. The best of p-values of those two tests were taken as a score for the given drug or compound. A multiple testing correction of the obtained p-values for the amount of compound perturbations under study was performed.

### Pathway-level similarity analysis

Pathway activation analysis is a powerful tool for extracting biologically-relevant properties from large transcriptomic datasets, enabling the generation of novel results prior to or in place of *in vitro* and *in vivo* experimentation. We have recently reported on a novel deep learning-based algorithm, the in silico Pathway Activation Network Decomposition Analysis (iPANDA), which we applied to large-scale trans-crip-tomic datasets as a tool for biomarker identification [104]. In contrast to other methods of pathway activation analysis, iPANDA generates pathway activation scores (PAS) by using precalculated gene coexpression data in combination with gene importance factors quantified according to the degree of differential gene expression and pathway topology decomposition. Here, we applied the same general approach to the task of drug mimicry, ranking existing nutraceutical compounds and compound combinations according to their transcriptomic signature's degree of similarity to the known transcriptomic signature of metformin and rapamycin.

For pathway-level similarity analysis we chose gene expression samples of drug induced transcriptional response from A549 cell line. Signaling pathway activation scores for 378 total pathways from SABiosciences collection (http://www.sabiosciences.com/pathwaycentral.php) were calculated for each perturbation of 871 natural compounds, including Metformin and Rapamycin, using iPANDA algorithm [104]. Similarity of two perturbations was measured as percent of commonly up- or down-regulated pathways between them.

Combination scoring. Additivity hypothesis was checked on the dataset E-MEXP-3192 (http://www.ebi.ac.uk/arrayexpress). Preprocessed gene expression data corresponding to samples that underwent 12 hour treatment with 100nm retinoic acid, 100 nm lapatinib and their combination was analysed with iPANDA algorithm. For several selected compounds pathway analysis was done for 97 age-related pathways [93]. PAS values for withaferin-A, ginsenoside, apigenin and GLA were measured in PC3 cells perturbed for 24 hours with 10μM of the compound with the exception of To estimate the combinatorial effect of 5 selected natural compounds with GRAS status PAS scores were summed for each combination of two or more compounds. Then we used Pearson correlation coef-ficient between metformin and the combination to estimate the similarity.

### Deep learning prediction of metformin/rapamycin signature and adverse effects

Deep neural networks (DNNs) were trained with transcriptional response data from the LINCS L1000 dataset. All available perturbations from MCF7, PC3, VCAP, A549, A375 and HT29 cell lines related to Metformin (perturbation id: BRD-K79602928) and Rapamycin (perturbation ids: BRD-A23770159, BRD-A50287119, BRD-A79768653, BRD-K84937637, BRD-K89626439, BRD-K99369265) were indepen-dently used and contributed to two training sets. Training and test sets were split at 80/20 ratio. For the Metformin prediction we used 67309 samples as the training set (98 samples are labeled as positive class) and 15788 samples as the test set (24 samples are labeled as positive class). For the Rapamycin prediction we used 68421 samples as the training set (517 samples are labeled as positive class) and 14677 samples as the test set (114 samples are labeled as positive class). The DNN was built by adjusting its hyperparameters (e.g. number of layers, activation function, etc.) on the training set and subsequently measuring the perfor-mance of the trained neural network on the test set. All experiments were conducted using an NVIDIA Titan X.

We used multilayer feed-forward neural networks as deep models (i.e. having more than 3 layers). Gridsearch algorithm was used for multiple hyper-parameters optimization in order to achieve the greatest predictive accuracy. We minimized the binary cross entropy loss function using a backpropagation algorithm. We used the Leaky ReLU activation function [192] in each layer, ADAM as optimizer of the cost function [193], dropout with 25% probability after each layer for the purposes of regularization [194] and additional L1 penalty on layer parameters.

Adverse effects for drugs were derived from SIDER database [83]. Side effect categories were mapped onto 321 preferred term from MedDRA v16.0 ontology [84]. An ensemble of class-specific DNNs with binary output was trained in a similar way to the methodology describ-ed previously [85]. All probabilities related to in a single side effect and perturbation id of the drug were aggregated.

## SUPPLEMENTARY MATERIAL


















