# Supplementary material for: Towards natural mimetics of metformin and rapamycin
Source: Aging (Albany NY). 2017 Nov 15;9(11):2245–68. doi: 10.18632/aging.101319 (PMC5723685; doi:10.18632/aging.101319)
Supplement: Supplementary file 9 [file aging-09-2245-s009.pdf]

SUPPLEMENTAL MATERIAL

Please browse the links in Full text version to see Supplementary Tables related to this manuscript.

Supplementary Table 1. Results of DNN classifier for Metformin.

Supplementary Table 2. Results of DNN classifier for Rapamycin.

Supplementary Table 3. Comparsion of all metformin or rapamycin perturbations to individual perturbations of other natural compounds on a gene level.

Supplementary Table 4. Comparsion of all metformin or rapamycin perturbations to individual perturbations of other natural compounds on a pathway level.

Supplementary Table 5. Pathway activation scores for Metformin and selected natural compounds.

Supplementary Table 6. Comparison of natural compounds combinations to Metformin on a pathway level.

Supplementary Table 7. Pathway-level similarity between metformin and four selected nutraceuticals in terms of pathway activation signature.

Supplementary Table 8. Predicted toxicity for each investigated compound (designated by pert_id and pert_desc columns). Mean probability across all adverse effects and the number of side effects with probability >0.5 were calculated and depicted in the table.

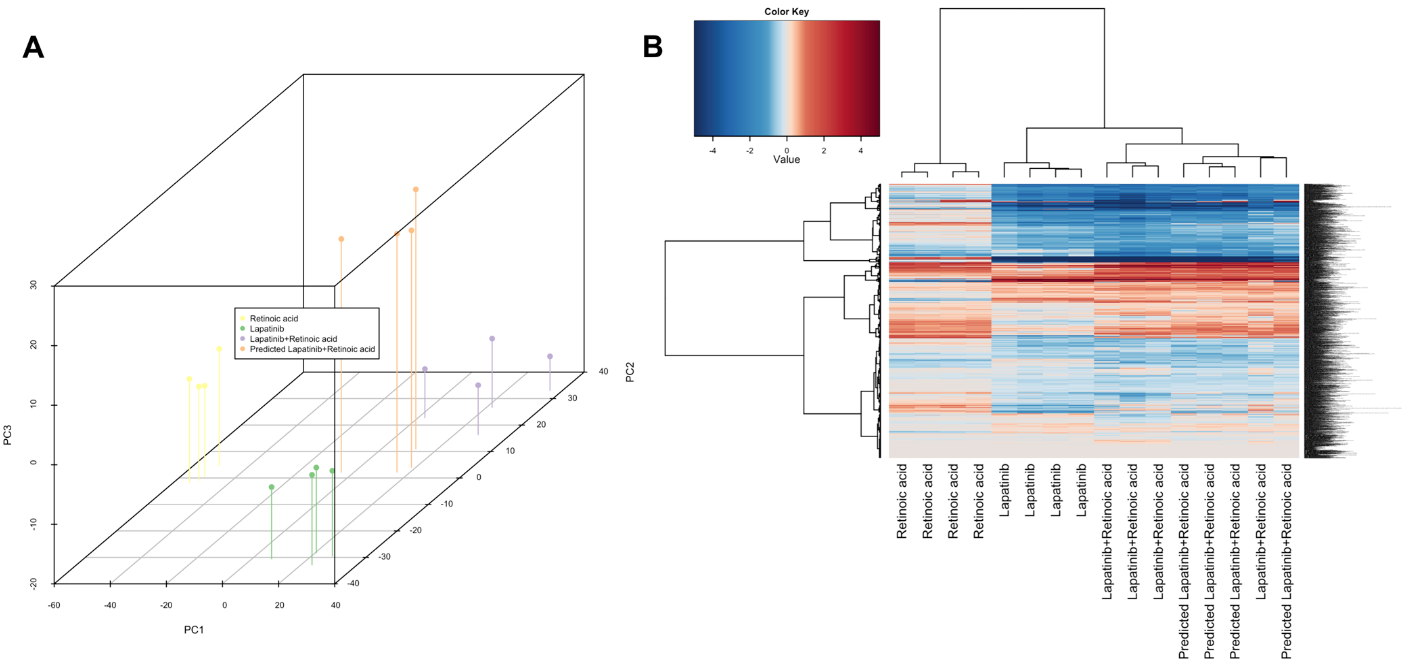

Supplementary Figure 1. Additivity analysis using E-MTAB-3192 dataset. PAS values are calculated independently for each perturbation of SKBR3 cells: 100nM Retinoic acid, 100nM Lapatinib and their combination. PCA analysis and heatmap demonstrate how close the predicted pathway perturbation of a combination is to the experimentally measured.

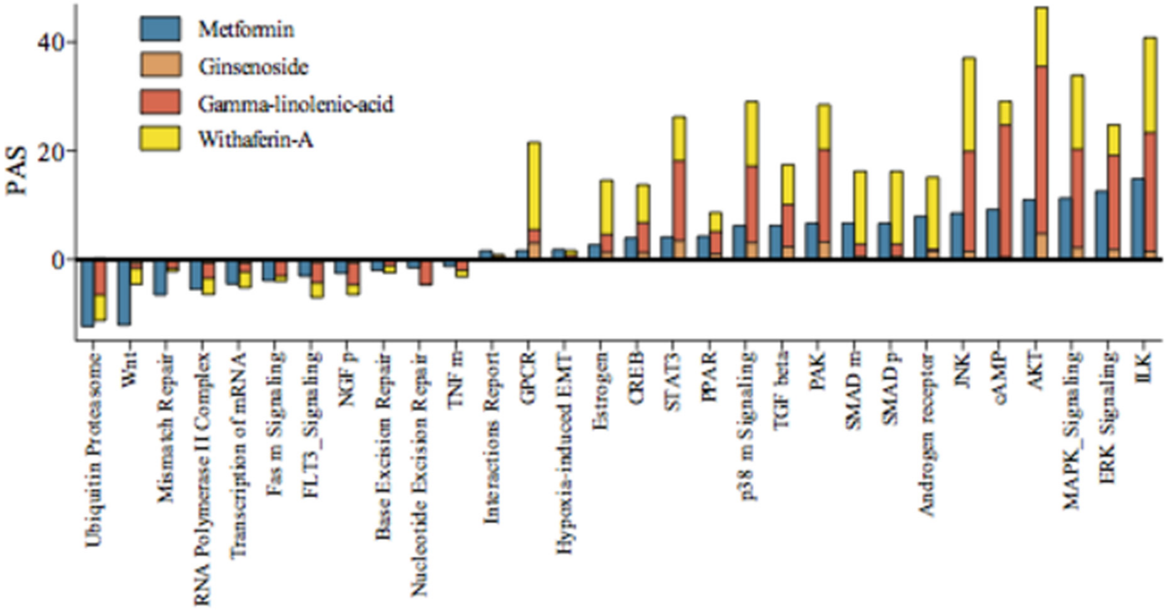

Supplementary Figure 2. Shared pathway activation between metformin and selected compound combination. Selected compounds for mimicking metformin included ginsenoside, gamma-linolenic acid, and withaferin-A. Here, metformin-activated or -inhibited aging pathways were selected, dependent on a metformin pathway activation strength (PAS) threshold of +/-1 and shared directionality of PAS for all three nutraceutical compounds comprising the selected combination. Predicted combination PAS for each pathway was defined as the sum of PAS for the individual nutraceuticals.
